# Prediction tool for discharge disposition and 30-day readmission using electronic health records among patients hospitalized for traumatic brain injury

**DOI:** 10.3389/fneur.2025.1581176

**Published:** 2025-06-16

**Authors:** Tianjian Zhou, James E. Graham, Davis Davalos-DeLosh, Amartya K. Maulik, Jessica Edelstein, Amanda L. Hoffman, Haonan Wang, Deana Davalos

**Affiliations:** ^1^Department of Statistics, Colorado State University, Fort Collins, CO, United States; ^2^Department of Occupational Therapy, Colorado State University, Fort Collins, CO, United States; ^3^Department of Computer Science, University of Colorado, Boulder, CO, United States; ^4^Shirley Ryan Ability Lab and Department of Physical Medicine & Rehabilitation, Northwestern University, Chicago, IL, United States; ^5^UCHealth, University of Colorado Hospital, Aurora, CO, United States; ^6^Department of Psychology, Colorado State University, CO, United States

**Keywords:** discharge planning, electronic health record, predictive modeling, readmission, rehabilitation, traumatic brain injury

## Abstract

**Background:**

Traumatic brain injury (TBI) is one of the most common and complex neurological conditions. Many TBI patients require ongoing rehabilitation beyond acute care, making treatment and discharge decisions critical. While individual risk factors for TBI outcomes are known, integrating comprehensive electronic health record (EHR) data into practical, validated prediction tools for personalized discharge planning and readmission risk assessment remains a key challenge. EHRs offer a valuable resource by integrating sociodemographic information, clinical care details, and prior healthcare encounters, providing an opportunity to develop models that predict key outcomes for TBI patients, such as discharge disposition and 30-day readmission.

**Methods:**

This retrospective cohort study utilized EHRs from a large multi-hospital health system (2017–2023) to develop and validate statistical models predicting discharge disposition and 30-day readmission among hospitalized TBI patients, and to translate these models into an accessible clinical prediction tool. Descriptive statistics were calculated to summarize patient characteristics. Multinomial logistic regression was used to model discharge disposition, and logistic regression was used for 30-day readmission. Forward stepwise regression based on the Akaike information criterion was used for variable selection. Cross-validation using the area under the receiver operating characteristic evaluated predictive performance.

**Results:**

Several factors were significantly associated with both outcomes. Older age was positively associated with discharge to Inpatient Rehabilitation Facility/Skilled Nursing Facility or Hospice/Died versus Home (*p* < 0.001), and with 30-day readmission (*p* = 0.002). Ethnicity, significant other status, insurance, prior inpatient stays, length of stay, as well as Glasgow Coma Scale, activities of daily living, and mobility were all significantly associated with discharge disposition (*p* < 0.001). Prior mental health diagnosis (*p* = 0.062), prior inpatient stays (*p* < 0.001), and intensive care unit admission (*p* = 0.002) were associated with higher odds of 30-day readmission, while Commercial insurance was associated with lower odds compared to Medicare (*p* = 0.024). A prediction tool is available.

**Conclusion:**

We developed and validated predictive models using EHR data, culminating in a practical tool that may enhance the management of patients hospitalized with TBI by supporting personalized discharge planning and risk stratification.

## 1 Introduction

Traumatic brain injury (TBI) is a prevalent neurological disorder associated with significant individual costs and a substantial societal burden worldwide (1). Recovery from TBI typically requires substantial time and resources, highlighting the importance of effective treatment planning to improve long-term outcomes and reduce complications. Given the finite availability of therapy staff and rehabilitation services (2), efficient resource allocation is essential. Predictive tools can help identify patients who may benefit from specific services, thereby supporting more targeted and patient-centered care. For example, identifying patients who are likely to require post-acute care in an inpatient rehabilitation facility (IRF) or skilled nursing facility (SNF) can facilitate early involvement of rehabilitation specialists, multidisciplinary care planning, and proactive communication with patients and families (3–5). Similarly, identifying patients with elevated risk of 30-day readmission can prompt targeted interventions such as medication reconciliation, focused education on warning signs, and timely post-discharge follow-up (6). Optimizing resource allocation, ensuring appropriate discharge disposition, and preventing readmissions all highlight the need for personalized treatment tailored to each patient’s prognostic profile. Historically, however, the ability to personalize patient care has been viewed as a luxury, often unattainable for resource-constrained acute care facilities (7).

Existing studies have explored predicting discharge disposition (8–11), 30-day readmission (12–15), and other outcomes, such as mortality and functional outcomes (16–20), among TBI patients. Nevertheless, a unique aspect of this study is its use of electronic health records (EHRs), a valuable resource for personalizing patient care. The widespread adoption of EHRs is a relatively recent development. In 2004, the United States government set a goal for all Americans to have an EHR by 2014 (21). However, it was not until the American Recovery and Reinvestment Act of 2009, which incentivized the adoption of EHRs, that many healthcare providers actively pursued the transition to EHR systems (22). Despite the widespread adoption of EHRs, they are sometimes perceived as a burden, diverting clinicians’ time and attention away from direct patient care (23). While various studies have focused on the challenges of EHRs, the unprecedented opportunities to leverage real-world clinical data to improve patient care are increasingly being recognized (24, 25). Still, translating the vast repository of real-world clinical data into reliable, interpretable, and actionable insights for specific conditions like TBI presents ongoing challenges. This study contributes to that effort by leveraging EHRs that contain a wealth of information on patient demographics, clinical care details, and medical history that influence recovery after TBI. Navigating these complex datasets and extracting meaningful insights necessitate advanced analytical techniques that can be translated into user-friendly tools.

Statistical predictive modeling is a powerful approach for analyzing complex healthcare data, identifying patterns and relationships that are not readily apparent to care providers, and creating personalized predictions of patient outcomes (26). These insights can inform clinical decision-making, such as treatment strategies and discharge planning, and help reduce readmission rates.

We aim to develop and validate statistical models using a comprehensive set of EHR-derived variables to predict two key patient outcomes following TBI hospitalization: discharge disposition and 30-day readmission. The goal is not only to identify significant predictors but also to transform these complex EHR data into an accessible prediction tool for clinicians, thereby demonstrating a practical pathway to support personalized treatment and discharge planning, optimizing resource allocation, and ultimately enhancing patient outcomes during and after acute care. The prediction tool can be accessed at https://tjzhou.shinyapps.io/INREACHapp/.

## 2 Materials and methods

### 2.1 Study design and setting

This retrospective cohort study analyzed data extracted from deidentified EHRs from a large multi-hospital health system. The study period encompassed records from 2017 to 2023. Data were obtained with support from Health Data Compass, an enterprise health data warehouse, and the study was approved by the Colorado Multiple Institutional Review Board and the Colorado State University Institutional Review Board.

### 2.2 Study population

Adult patients (18 years or older) hospitalized with a primary diagnosis of TBI were considered for inclusion. TBI diagnoses were identified using the International Classification of Diseases, Tenth Revision (ICD-10) codes.

### 2.3 Data collection and preprocessing

**Figure 1:**
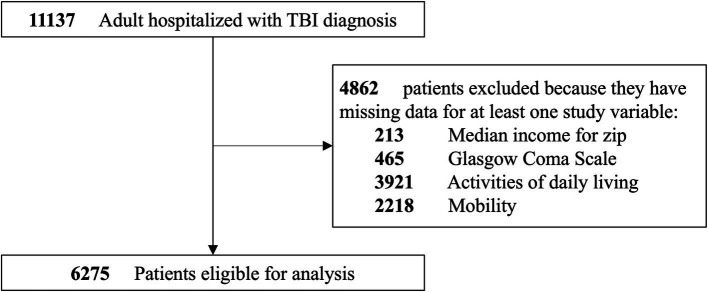
Cohort selection diagram. (i) There can be overlapping missing data (e.g., both GCS and ADL can be missing for the same patient); (ii) when GCS, ADL, and mobility are missing, it is always the case that both the minimum and maximum scores are missing.

Data were extracted from the EHR system, including patient demographics, medical histories, treatment details, and outcomes. The initial dataset of 11,137 patients was cleaned and coded, resulting in a final analytical cohort of 6,275 patients (Figure 1). A total of 4,862 patients were excluded because they had missing data for at least one of the following continuous variables: median income for zip code (*n* = 213), Glasgow coma scale (GCS, *n* = 465), activities of daily living (ADL, *n* = 3,921), and mobility (*n* = 2,218). Some patients had more than one missing variable. Missing data for categorical variables were handled by creating a separate “Missing” category.

### 2.4 Predictor variables

A comprehensive set of predictor variables was considered, including:

- **Sociodemographic factors**: Age, race, ethnicity, sex, significant other status, median income for zip code, percentage above high school education for zip code, and percentage above bachelor’s education for zip code.
- **Substance use**: Tobacco, alcohol, and drug use.
- **Health and functional status**: Glascow Coma Scale (GCS), a clinical tool used to assess a patient’s level of consciousness by evaluating verbal, eye, and motor responses (27, 28). The GCS score ranges from 3 to 15, with higher scores indicating higher levels of responsiveness. Activities of daily living (ADL), measured using the Activity Measure for Post-Acute Care (AM-PAC) “6-Clicks” tool, an electronically administered questionnaire (29, 30). The ADL score evaluates self-care abilities and ranges from 6 to 24, with higher scores indicating greater self-care independence. Mobility, also measured using the AM-PAC “6-Clicks.” The mobility score evaluates a patient’s ability to perform mobility tasks and ranges from 6 to 24, with higher scores reflecting greater mobility function. During a patient’s stay, multiple measurements of GCS, ADL, and mobility were taken, and the minimum and maximum scores recorded from assessments conducted throughout their acute care hospitalization were used. This approach captures the observed range of a patient’s neurological and functional status during their admission, rather than relying on a single time-point (e.g., initial emergency department GCS) assessment, to inform predictions related to discharge planning and 30-day readmission risk from the perspective of the entire hospital course.
- **Hospital encounter information**: Total length of stay, emergency department (ED) visits, and intensive care unit (ICU) admission.
- **Insurance and payment information**: Insurance type (Medicare, Medicaid, Commercial, and Self-pay/Other).
- **Prior medical history**: Prior mental health diagnosis and inpatient stays.

### 2.5 Outcome variables

The primary outcome variables of interest were:

- **Discharge disposition**: Categorized as Home, IRF/SNF, Hospice/Died, and Other.
- **30-day readmission**: Defined as any unplanned hospital readmission within 30 days of the initial discharge date for TBI.

### 2.6 Statistical analysis

Descriptive statistics, including frequencies for categorical variables and means with standard deviations for continuous variables, were calculated to summarize patient characteristics. Multinomial logistic regression was used to model discharge disposition (a categorical variable), with the reference category for the outcome variable set to “Home” (31). Logistic regression was used to model 30-day readmission (a binary variable) (31). Forward stepwise regression based on the Akaike information criterion (AIC) was employed for variable selection and to identify important predictors for each outcome, with a maximum of 10 predictors included in each final model for ease of interpretation and model parsimony (32–34). A reasonable reference category was selected for each categorical predictor. Likelihood ratio tests were used to calculate *p*-values from multiple regression models that included all important predictors identified through stepwise selection. Statistical significance was determined using a *p*-value threshold of 0.05. Additionally, *p*-values from individual simple regression models with one predictor at a time (marginal *p*-values) were also calculated and reported. This approach captures both the conditional effect, the significance of a predictor after controlling for the other important predictors, and the marginal effect, which reflects a predictor’s significance when considered independently from the other predictors. The *p*-values are intended for descriptive purposes, with the understanding that their interpretation is complicated by post-selection inference (35) and multiple testing (36).

The area under the receiver operating characteristic curve (AUC) was used to evaluate the predictive performance of forward stepwise logistic regression (37). When the outcome variable has more than 2 classes (discharge disposition), the multiclass AUC was applied (38). To better capture out-of-sample predictive performance, 5-fold cross-validation was performed (39). Other candidate models and variable selection methods, including random forest (40), the least absolute shrinkage and selection operator (LASSO) (41), support vector machine (SVM) (42), and the full logistic regression model without variable selection, were compared based on cross-validated AUC values. Note that random forest, LASSO, and SVM do not provide straightforward quantification of predictor significance through *p*-values.

The R statistical software was used to fit the models to the data (43). The multinomial logistic regression model was fitted using the multinom function from the nnet package (44). Logistic regression was performed with the glm function in base R. Forward stepwise regression, based on the AIC, was conducted using the step function in base R. AUC values were calculated with the pROC package (45). The random forest model was fitted with the randomForest package (46), while the LASSO method was implemented using the glmnet package (47). The SVM method was implemented using the svm function from the e1071 package (48). The shiny package was used to create an R Shiny app that predicts a patient’s discharge disposition and chance of 30-day readmission (49).

## 3 Results

### 3.1 Study population characteristics

The study cohort comprised 6,275 adult patients hospitalized for TBI. Descriptive statistics are provided in Tables 1, 2. The mean age was 60.74 years (SD = 20.82), and 60.41% of the patients were male. The racial distribution of the cohort was as follows: 78.47% White, 5.40% Black, 1.91% Asian, 0.76% Native American, 0.29% Pacific Islander, 0.62% Multiple Race, and 12.54% Missing. The ethnic distribution of the cohort was: 84.62% Non-Hispanic, 14.15% Hispanic, and 1.23% Missing. The distribution of insurance types was: 49.72% Medicare, 20.13% Medicaid, 20.41% Commercial, and 9.74% Self-pay/Other. Prior mental health conditions were present in 10.61% of the cohort, and 16.03% of the cohort had prior inpatient stays. The average length of stay was 11.48 days (SD = 18.51), and 55.90% of patients required an ICU admission. 67.73% of patients had an ED visit, 7.01% did not have an ED visit, and for the remaining 25.26%, their ED visit status was missing. The cohort’s mean minimum and maximum GCS scores during their stays were 11.87 (SD = 4.14) and 14.83 (SD = 0.68), respectively. The mean minimum and maximum ADL scores were 15.24 (SD = 5.31) and 17.55 (SD = 4.78), while the mean minimum and maximum mobility scores were 13.94 (SD = 5.48) and 18.34 (SD = 4.75). The distribution of discharge disposition of the cohort was: 50.57% Home, 38.74% IRF/SNF, 3.68% Hospice/Died, and 7.01% Other. Lastly, 8.61% of the patients had unplanned 30-day readmission.

**Table 1:** Descriptive statistics of discrete variables for the study population (*n* = 6,275).

Variable	Category	Frequency (%)
Sociodemographic factors		
Sex	Male	3,791 (60.41%)
	Female	2,484 (39.59%)
Race	White	4,924 (78.47%)
	Black	339 (5.40%)
	Asian	120 (1.91%)
	Native American	48 (0.76%)
	Pacific Islander	18 (0.29%)
	Multiple Race	39 (0.62%)
	Missing	787 (12.54%)
Ethnicity	Non-Hispanic	5,310 (84.62%)
	Hispanic	888 (14.15%)
	Missing	77 (1.23%)
Significant other	No	3,146 (50.14%)
	Yes	2,615 (41.67%)
	Missing	514 (8.19%)
Substance use		
Tobacco	No	4,036 (64.32%)
	Yes	1854 (29.55%)
	Missing	385 (6.14%)
Alcohol	No	2,426 (38.66%)
	Yes	3,422 (54.53%)
	Missing	427 (6.80%)
Drug	No	4,360 (69.48%)
	Yes	1,403 (22.36%)
	Missing	512 (8.16%)
Hospital encounter information		
ED visit	No	440 (7.01%)
	Yes	4,250 (67.73%)
	Missing	1,585 (25.26%)
ICU admission	No	2,767 (44.10%)
	Yes	3,508 (55.90%)
Insurance and payment information		
Insurance	Medicare	3,120 (49.72%)
	Medicaid	1,263 (20.13%)
	Commercial	1,281 (20.41%)
	Self-pay/Other	611 (9.74%)
Prior medical history		
Prior mental health diagnosis	No	5,609 (89.39%)
	Yes	666 (10.61%)
Prior inpatient stays	No	5,269 (83.97%)
	Yes	1,006 (16.03%)
Outcome variables		
Discharge disposition	Home	3,173 (50.57%)
	IRF/SNF	2,431 (38.74%)
	Hospice/Died	231 (3.68%)
	Other	440 (7.01%)
30-day readmission	No	5,735 (91.39%)
	Yes	540 (8.61%)

**Table 2:** Descriptive statistics of continuous variables for the study population (*n* = 6,275).

Variable	Range	Mean (SD)
Sociodemographic factors		
Age	18—103 Years	60.74 (20.82)
Median income for zip	11,000—203,000 USD	69028.23 (22493.28)
% High school for zip	4.80—93.40%	33.27 (12.20)
% Bachelor’s for zip	0.30—88.40%	9.58 (5.44)
Health and functional status		
Glasgow Coma Scale (Min)	3—15	11.87 (4.14)
Glasgow Coma Scale (Max)	4—15	14.83 (0.68)
Activities of daily living (Min)	6—24	15.24 (5.31)
Activities of daily living (Max)	6—24	17.55 (4.78)
Mobility (Min)	6—24	13.94 (5.48)
Mobility (Max)	6—24	18.34 (4.75)
Hospital encounter information		
Total length of stay	0—413 Days	11.48 (18.51)

### 3.2 Discharge disposition model

Multinomial logistic regression was used to model discharge disposition, with “Home” set as the reference category for the outcome variable. Forward stepwise regression was employed to identify 10 predictors that were most predictive of discharge disposition. Table 3 shows the full model output with regression coefficients, *p*-values, and marginal *p*-values. Older age, prior inpatient stays, and longer length of stay were all significantly associated with a greater likelihood of discharge to IRF/SNF, Hospice/Died, or Other compared to Home (all *p* < 0.001). Being Hispanic, having a significant other, having Medicaid or Commercial insurance rather than Medicare, higher minimum GCS scores, higher maximum ADL scores, and higher minimum/maximum mobility scores were significantly associated with a greater likelihood of being discharged Home compared to all other categories (all *p* < 0.001). Higher maximum GCS score was linked to a lower likelihood of discharge to Hospice/Died and Other compared to Home, but a greater likelihood to IRF/SNF (*p* < 0.001). Higher minimum ADL score was linked to a lower likelihood of discharge to IRF/SNF and Other compared to Home, but a greater likelihood to Hospice/Died (*p* < 0.001). The difference in discharge disposition between patients with and without an ED visit was not significant when controlling for the above predictors (*p* = 0.211). However, ED visit was selected by forward regression, driven primarily by the “Missing” category (*p* = 0.003).

**Table 3:** Multinomial logistic regression results for discharge disposition.

Predictor	IRF/SNF	Hospice/Died	Other	*p*-value	Marginal *p*-value
Intercept	2.531	5.406	8.717		
Age	0.018	0.054	0.007	< 0.001	< 0.001
Ethnicity				< 0.001	< 0.001
Non-Hispanic	.	.	.		
Hispanic	−0.469	−0.623	−0.694	< 0.001	< 0.001
Missing	0.145	1.146	0.755	0.129	0.020
Significant other				< 0.001	< 0.001
No	.	.	.		
Yes	−0.399	−0.229	−0.217	< 0.001	0.008
Missing	−0.180	0.691	−0.132	0.004	< 0.001
Insurance				< 0.001	< 0.001
Medicare	.	.	.		
Medicaid	−0.953	−0.695	−0.208	< 0.001	< 0.001
Commercial	−0.524	−0.942	−0.159	< 0.001	< 0.001
Self-pay/Other	−0.742	−0.729	0.048	< 0.001	< 0.001
Glasgow Coma Scale				< 0.001	< 0.001
Min	−0.024	−0.197	−0.137	< 0.001	< 0.001
Max	0.277	−0.096	−0.140	< 0.001	< 0.001
Activities of daily living				< 0.001	< 0.001
Min	−0.051	0.036	−0.008	< 0.001	< 0.001
Max	−0.135	−0.304	−0.211	< 0.001	< 0.001
Mobility				< 0.001	< 0.001
Min	−0.087	−0.125	−0.001	< 0.001	< 0.001
Max	−0.150	−0.139	−0.186	< 0.001	< 0.001
Prior inpatient stays				< 0.001	< 0.001
No	.	.	.		
Yes	0.087	0.845	0.114	< 0.001	< 0.001
ED visit				0.006	< 0.001
No	.	.	.		
Yes	−0.057	0.481	−0.181	0.211	0.007
Missing	−0.190	0.624	−0.597	0.003	< 0.001
Length of stay	0.021	0.025	0.023	< 0.001	< 0.001

The “Home” discharge setting is chosen as the reference category for the outcome variable, with the other categories, “IRF/SNF,” “Hospice/Died,” and “Other,” compared against “Home.” Important predictors are selected by forward stepwise regression. A “.” in the regression coefficient indicates that the category is designated as the reference category for the corresponding predictor variable.

### 3.3 30-day readmission model

Logistic regression was used to model 30-day readmission, with forward stepwise regression identifying 7 predictors that were most predictive of the outcome variable. The full model results, including regression coefficients, *p*-values, and marginal *p*-values, can be found in Table 4. Older age (*p* = 0.002) and prior inpatient stays (*p* < 0.001) were significantly associated with higher odds of readmission. Commercial insurance was associated with lower odds of readmission compared to Medicare (*p* = 0.024), while the difference between Medicaid and Medicare was not significant (*p* = 0.963). Prior mental health diagnosis, being marginally significant in the multiple regression model (*p* = 0.062), was associated with higher odds of readmission. ICU admission, significant only in the multiple regression model (*p* = 0.002), was also associated with higher odds of readmission. The difference in 30-day readmission between patients with and without a significant other (*p* = 0.716) and with and without alcohol use (*p* = 0.571) was not significant when controlling for the other predictors included in the model. However, significant other status and alcohol use were selected by forward regression, driven primarily by the “Missing” category (*p* < 0.001 and *p* = 0.006, respectively).

**Table 4:** Logistic regression results for 30-day readmission.

Predictor	Regression coef.	*p*-value	Marginal *p*-value
Intercept	−3.310		
Age	0.011	0.002	< 0.001
Significant other		< 0.001	< 0.001
No	.		
Yes	0.035	0.716	0.470
Missing	−1.167	< 0.001	< 0.001
Alcohol		0.004	< 0.001
No	.		
Yes	0.054	0.571	0.772
Missing	−0.908	0.006	< 0.001
Insurance		0.064	< 0.001
Medicare	.		
Medicaid	0.008	0.963	< 0.001
Commercial	−0.379	0.024	< 0.001
Self-pay/Other	−0.152	0.441	< 0.001
Prior mental health diagnosis		0.062	< 0.001
No	.		
Yes	0.288	0.062	< 0.001
Prior inpatient stays		< 0.001	< 0.001
No	.		
Yes	0.649	< 0.001	< 0.001
ICU admission		0.002	0.408
No	.		
Yes	0.296	0.002	0.408

Important predictors are selected by forward stepwise regression. A “.” in the regression coefficient indicates that the category is designated as the reference category for the corresponding predictor variable.

### 3.4 Model evaluation

The AUC was used as the performance metric to assess the predictive performance of the forward stepwise logistic regression model (37, 38). The AUC quantifies the model’s ability to distinguish between outcome categories by integrating sensitivity and specificity, providing a summary of performance across all classification thresholds. This makes the AUC particularly useful for applications involving imbalanced outcomes, as in this study. The AUC ranges from 0.5 to 1, with higher values indicating stronger discriminatory power. To obtain a robust estimate of out-of-sample performance, 5-fold cross-validation was employed. The data were randomly partitioned into five subsets, with the model trained on four subsets and tested on the remaining one. This process was repeated five times, with each subset serving as the test set once, and the average AUC value was taken.

For comparison, additional candidate models and variable selection methods were considered, including random forest, LASSO, SVM, and the full logistic regression model without variable selection. These models were selected to represent a range of approaches varying in complexity and interpretability. Their performance was compared to the stepwise logistic regression model.

The cross-validated AUC values for each model are presented in Table 5. For discharge disposition, the forward stepwise logistic regression model achieved an AUC of 0.773, comparable to the AUC of 0.779 for random forest, 0.770 for LASSO, and 0.772 for the full model. SVM yielded a slightly lower AUC of 0.759. For 30-day readmission, the forward stepwise logistic regression model achieved an AUC of 0.656, comparable to LASSO (AUC = 0.655) and outperforming random forest (AUC = 0.609), SVM (AUC = 0.546), and the full model (AUC = 0.638).

**Table 5:** 5-fold cross-validated AUC for measuring and comparing the predictive performance of forward stepwise logistic regression (Forward), random forest (RF), the least absolute shrinkage and selection operator (LASSO), support vector machine (SVM), and the full logistic regression model without variable selection (Full).

Outcome	Forward	RF	LASSO	SVM	Full
Discharge disposition	0.773	0.779	0.770	0.759	0.772
30-day readmission	0.656	0.609	0.655	0.546	0.638

Based on these results, the forward stepwise logistic regression model demonstrated good predictive performance for both discharge disposition and 30-day readmission, comparable to or better than the alternative models considered. Given its competitive performance, straightforward variable selection, and the added benefit of providing interpretable regression coefficients and *p*-values, forward stepwise logistic regression was selected as the primary modeling approach for this study.

### 3.5 Prediction tool

An R Shiny app was developed to provide an interactive interface for these predictive models. The app allows clinicians to input a patient’s values for the identified predictors and subsequently receive estimated probabilities for different discharge dispositions and the likelihood of 30-day readmission. This tool aims to support clinical decision-making for treatment and discharge planning by predicting a patient’s discharge disposition and chance of 30-day readmission. The app is available at https://tjzhou.shinyapps.io/INREACHapp/.

## 4 Discussion

We used statistical predictive modeling to create a practical tool that may support treatment and discharge planning for TBI patients, leveraging the rich data available in EHRs. While several factors identified in our models, such as age and GCS, are established predictors of TBI outcomes (9, 12, 18), this study’s primary contribution extends beyond re-identifying individual risk factors. We demonstrate the utility of integrating a *comprehensive suite of readily available EHR* var*iables,* encompassing sociodemographics, functional status, hospital encounter details, insurance, and medical history from a large, multi-hospital system into parsimonious yet robust predictive models. Furthermore, we show that forward stepwise logistic regression, a highly interpretable method, performs comparably or better than more complex machine learning approaches like random forests or SVMs for these specific TBI outcomes (Table 5). Crucially, we translate these validated models into an *accessible, interactive prediction tool*, bridging the gap between data analysis and potential clinical application. Our analyses identified several important predictors for discharge disposition and 30-day readmission, providing valuable insights for clinical decision-making.

The INREACHapp prediction tool^1^ is designed to translate these statistical insights into actionable information at the point of care. For instance, when a clinician inputs a patient’s data, the tool provides probabilities for discharge to Home, IRF/SNF, Hospice/Died, or Other. A high predicted probability for IRF/SNF might prompt earlier engagement of rehabilitation specialists, multidisciplinary team discussions about post-acute care needs, and proactive communication with the patient and family regarding expectations and planning. Similarly, a high predicted risk of 30-day readmission could trigger targeted interventions. These might include comprehensive medication reconciliation, enhanced patient and caregiver education focused on warning signs, scheduling prompt post-discharge follow-up appointments, or coordinating with community-based services to ensure a smoother care transition. The current models utilize summary variables from the hospital stay (e.g., length of stay, min/max functional scores) and are thus most relevant for informing discharge planning as the patient stabilizes. While the tool can be used whenever these data points are available, dynamic, day-to-day prediction based on evolving patient status would represent a future development, potentially requiring different predictors and model structures.

Regarding the discharge disposition model, several factors were predictive of discharge to different settings. Not unexpectedly, older age was associated with a higher likelihood of discharge to IRF/SNF, Hospice/Died, or Other rather than home. Similarly, a history of prior inpatient stays and a longer length of stay were significantly associated with a higher likelihood of discharge to other settings instead of home. This is not surprising, as prior hospitalizations and extended stays likely indicate a more complex medical history and greater symptom severity, necessitating continued post-discharge care. The inclusion of both minimum and maximum functional scores (GCS, ADL, Mobility) by the stepwise selection process suggests that both the lowest point of function and the peak recovery achieved during hospitalization contribute unique predictive information for discharge disposition.

In contrast, identifying as Hispanic, having a significant other, being insured through Medicaid or Commercial insurance rather than Medicare, and having higher minimum GCS, maximum ADL, and minimum/maximum mobility scores were all significantly associated with a higher likelihood of being discharged home rather than to other settings. Factors reflecting less severe brain injury (GCS) and improved health and mobility (ADL and mobility) are logically related to a greater likelihood of home discharge. It is important to interpret the discharge disposition model outputs with nuance. The “Home” category was used as the reference in our multinomial logistic regression, a standard statistical approach, and its prediction does not inherently imply it is a “better” outcome than IRF/SNF for every individual. The most appropriate discharge setting is a complex clinical decision tailored to individual patient needs, functional status, and support systems. Our model aims to predict the observed discharge patterns within our healthcare system based on the available data, thereby providing insights into factors influencing these decisions and helping to anticipate post-acute care needs and resource utilization. The primary outcomes modeled were the discharge event itself and 30-day readmission, not comparative long-term functional outcomes across different discharge settings, which would necessitate different study designs and outcome measures.

The association between identifying as Hispanic and discharge disposition may be influenced by various factors, as discussed previously (50–54). Our findings regarding ethnicity’s association with discharge disposition, even within a cohort that was predominantly Caucasian, highlight the complex interplay of socio-cultural factors in healthcare decisions and outcomes. This underscores the need for future research to validate and potentially recalibrate such predictive models in more racially and ethnically diverse TBI populations to ensure their equitable applicability and to uncover population-specific predictors. Similarly, the presence of a significant other facilitating home discharge aligns with prior research (55), though the complexities noted (56) remain relevant.

In terms of 30-day readmission, factors such as older age, insurance status, prior mental health diagnosis, previous inpatient stays, and ICU admission were associated with a higher risk. Readmission after acute care for TBI is not merely an indicator of care quality or a source of financial strain for patients and healthcare systems (57, 58); it often signals unresolved medical issues or the emergence of TBI-related complications such as post-traumatic seizures, persistent headaches, or neurological decline. Such early readmissions can negatively impact long-term functional recovery, increase overall morbidity, and are associated with a higher risk of mortality (13, 57, 59–61). While our model identified factors like prior inpatient stays and ICU admission as significant predictors or proxies for greater medical complexity and severity, specific TBI sequelae (e.g., seizure disorders, persistent headaches) or detailed mechanisms of injury were not available as discrete variables for inclusion in this particular dataset. Future iterations of predictive models could be enhanced by incorporating such granular clinical information where feasible, potentially improving predictive accuracy for TBI-specific adverse events.

Many of our findings regarding individual predictors are consistent with previous studies (9, 12, 18). However, our study expands upon prior research by leveraging EHR data from a large multi-hospital health system, which capture a broader range of predictors simultaneously. This allows us to develop a more comprehensive predictive model using interpretable statistical methods that perform comparably to more complex machine learning techniques in this context.

Several directions can be pursued to further validate and generalize the current study. First, applying the proposed method to other sources of EHR data can help assess the robustness of the conclusions. Second, the method remains applicable even when predictions are made at different time points, with candidate predictors added or dropped based on data availability. Third, developing a flexible software infrastructure that can adapt to varying EHR structures and automate the construction of the prediction tool across different datasets would enhance its scalability and usability. Fourth, incorporating unstructured EHR data could make the predictive model more comprehensive (62). Fifth, expanding the tool to include other relevant outcomes would further increase its clinical utility. Lastly, evaluating the feasibility and impact of integrating the prediction tool into clinical workflows is a critical next step.

The principles and methodologies central to this study, which include the systematic leveraging of comprehensive EHR data, the application of robust statistical predictive modeling, and the development of user-friendly decision-support tools, hold considerable potential for broader application in TBI care worldwide. While specific predictor variables and their weights will undoubtedly vary across different healthcare systems, patient demographics, and cultural contexts, the foundational approach of transforming routinely collected clinical data into actionable, personalized insights can empower clinicians globally. This can lead to more efficient resource allocation, timely interventions for at-risk patients, and ultimately, contribute to improving TBI care pathways and outcomes. Future international collaborations could focus on standardizing key TBI-related data elements within EHRs and sharing best practices for the development, validation, and ethical implementation of such predictive tools across diverse settings.

## 5 Limitations

This study has several strengths, including a large sample size from a multi-hospital system, the use of real-world EHR data, rigorous statistical methodology, and the development of a user-friendly app. However, certain limitations should be acknowledged. As a retrospective cohort study, there is potential for selection bias and unmeasured confounding (63). Therefore, the findings should be interpreted as associations that do not necessarily represent causal relationships. The study cohort, while large, was predominantly Caucasian (78.47% White). This may limit the generalizability of the specific model coefficients to TBI populations with different racial and ethnic compositions. Future validation and calibration in more diverse cohorts are essential. Certain factors potentially pertinent to TBI outcomes, such as the detailed mechanism of injury or the presence of specific post-TBI complications (e.g., seizures, neurobehavioral symptoms not captured by broader mental health diagnoses), were not available as discrete, structured variables in our dataset for model inclusion. Their absence might affect the model’s comprehensiveness (11, 13). The GCS scores utilized (minimum and maximum during hospitalization) differ from the initial ED GCS often reported in acute TBI prognostic studies. While these scores reflect a patient’s trajectory during admission and are relevant for discharge planning, they may capture different aspects of neurological status than a one-time ED assessment. Missing data, a common challenge in EHR analyses, were handled by excluding records with missing values in continuous variables and creating a “Missing” category for categorical variables with missingness. However, the impact of missing data on the findings requires further investigation, as nonignorable missingness may introduce bias. For example, underserved groups may be more susceptible to missing data due to fragmented care or language barriers (64). Additionally, the “Missing” category in several categorical variables, including ethnicity, significant other status, ED visits, and alcohol use, was significantly associated with discharge disposition and 30-day readmission (Tables 3, 4). Given the inherent ambiguity of the “Missing” category, it is unclear whether it reflects specific patient characteristics, underlying conditions, or reporting trends at certain facilities. As a result, findings related to this category should be interpreted with caution. Moreover, while ethnicity was found to be significantly associated with discharge disposition, race was not identified as an important predictor for either outcome. This may be due to the small number of participants identifying as a race other than White or Missing (Table 1). Lastly, the models developed in this study estimate the likelihood of various discharge dispositions and the chance of 30-day readmission based on the patterns in the EHR data. These predictions reflect overall trends and should be used in conjunction with clinical judgment, not to replace clinical judgment and holistic patient assessment, ensuring that all patients receive individualized care based on their comprehensive needs.

## Data Availability

The datasets presented in this article are not readily available because the data analyzed in this study were obtained through a data use agreement with the University of Colorado Hospital Authority (UCHA). Others must establish their own data use agreements with UCHA to access these datasets. Requests to access the datasets should be directed to https://www.uchealth.org/locations/uchealth-university-of-colorado-hospital-uch/.

## Footnotes

^1^ https://tjzhou.shinyapps.io/INREACHapp/
